# Massive lower gastrointestinal bleeding attributable to heavy whipworm infection and *Salmonella typhi* co-infection: a case report

**DOI:** 10.4076/1757-1626-2-8285

**Published:** 2009-09-16

**Authors:** Amal Rashad Nimir, Mohamad Salleh Bin Abdul Aziz, Geok Chin Tan, Abdul Rahman Hikmet Shaker

**Affiliations:** 1 Department of Parasitology & Entomology, Universiti KebangsaanMalaysia; 2 Department of Surgery, Hospital Universiti KebangsaanMalaysia; 3 Department of Pathology, Hospital Universiti KebangsaanMalaysia

## Abstract

Massive lower gastrointestinal bleeding due to trichuriasis and/or typhoid fever is rarely reported. We reported a case of a 29-year-old male presented with per rectal bleeding, diarrhea, generalized abdominal pain and fever for two weeks. After diagnosis suspicion, emergency exploratory laparotomy was performed, where resection of the ulcerated part of the caecum and terminal ileum was performed. Microscopically analysis, diagnosed heavy infestation with *Trichuris trichiura*. It was complicated with *Salmonella typhi* infection confirmed later from the blood culture result.

## Introduction

Life threatening lower GI bleeding is very rare since it is usually intermittent and self-limiting. It can be caused by inflammation, neoplasm, anatomical abnormalities and infection caused by different pathogens.

Trichuriasis is infection of the large intestine caused by the human whipworm *Trichuris trichiura*. This nematode was found to be the commonest soil-transmitted helminthes among Aborigine children in Malaysia. It was mostly observed in the age group of 2-7 years, where incidence of pica is the highest [1,2] added to this; the hot, moist climate and poor sanitation which favor the worms survival.

Infection is acquired through ingestion of parasitic ova. Patients usually present with dysentery, rectal prolapse, hypochromic anemia, abdominal pain and growth retardation. Chronic cases may also present with hypoproteinaemic oedema and cardiac failure due to severe anemia.

Although the incident rate of typhoid and paratyphoid is decreasing in Malaysia [3], but still we have few cases admitted in our hospital in year 2008. Majority of patients in affluent countries present with the triad of persistent fever, headache and abdominal symptoms.

Abdominal examination may reveal hepatomegaly, splenomegaly and diffused abdominal tenderness. In more serious cases gaseous abdominal distention occurs and this may herald the onset of the acute abdomen that accompanies ileal perforation. Complications of typhoid infection are bleeding, perforation, circulatory collapse and relapse following treatment. We report this case as rarity and misleading diagnosis of the underlying cause of lower GI bleeding.

## Case presentation

**Figure 1.**
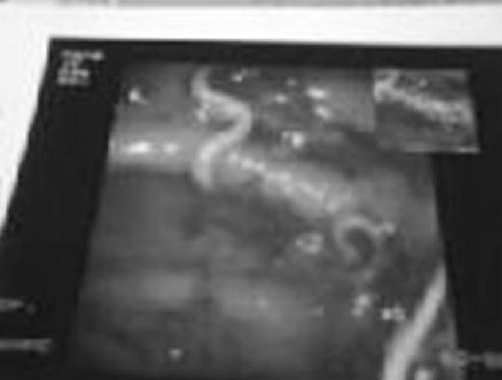
Whipworm, multiple ulcers and blood clots in the ileum.

**Figure 2.**
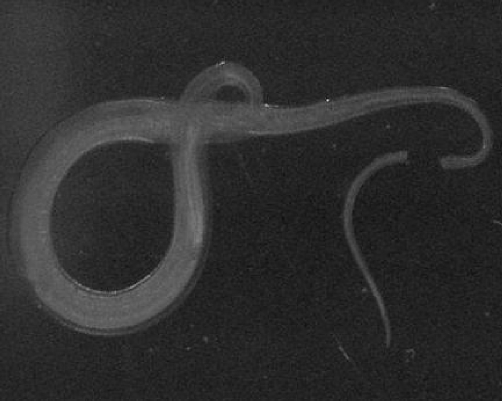
Adult worm obtained from the excised intestinal part.

**Figure 3.**
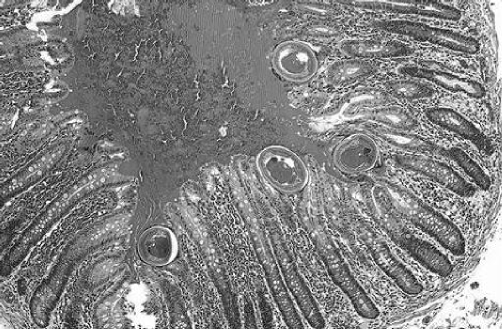
Section of the colon showing multiple mucosal ulcerations with presence of whip worm burrows into the mucosa and blood clots in the intestinal lumen.

**Figure 4.**
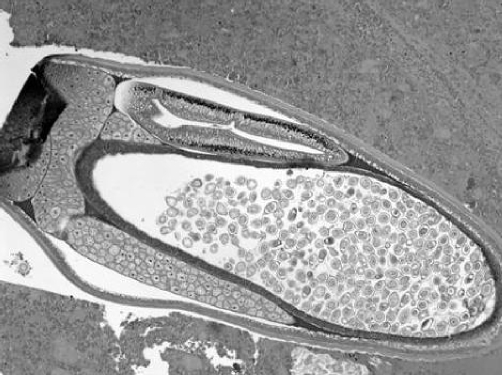
Some of the parasites contain numerous eggs.

A 29-year-old Bangladesh man was admitted to Hospital Universiti Kebangsaan Malaysia, complaining of per rectum bleeding. He gave history of diarrhea and generalized abdominal pain accompanied by fluctuating fever for 2 weeks. Personal and family history had not relevant information. On physical examination, the patient was dehydrated, pale, hypotensive (BP = 90/50) with accelerated heart rate (PR = 117/min). Digital rectal examination preceded with proctoscope showed fresh blood. Full blood count results, renal profile and liver function test are presented in Table 1. Upper GI scope showed ulcers at lesser curvature, patient was diagnosed as having gastric ulcer that was secured with adrenaline injection. The patient developed hypovolemic shock 6 hours post procedure. After giving him fleet enema, a complete colonoscopy up to terminal ileum was performed. Multiple bleeding ulcers in the caecum and terminal ileum were seen in addition to overwhelming of the intestinal lumen with small white worms. One cycle of DIVC regime and 4 pint of packed cell was transfused along; Albendazole 400 mg once daily for 3 days was prescribed. Two hours later, patient had another bleeding episode and emergency exploratory laparotomy was performed. On table enteroscopy revealed overwhelming of the bowels with whipworm, multiple ulcers, blood clots in the caecum and ileum (Figure 1). The excised intestinal part was sent to Pathology and Parasitology laboratories. Results from both sides confirmed heavy infection with *T. trichiura* (Figures 2,3 & 4). Patient had persistent fever with leucopaenia and thrombocytopaenia few days postoperative. Blood culture was done at that time to reveal a positive *S. typhi* infection. With one course of intravenous ciprofloxacin given, fever subsides and no more episode of lower GI hemorrhage. Patient discharged well, with negative results for both fecal specimen and blood culture.

**Table 1.** Laboratory investigations results

Test	Results	Units	Range
**Full Blood Count**			
White Cell Count	4.4	×10 ^9/L	(4.0-10.0)
Red Cell Count	**2.98**	×10 ^12/L	(3.8-5.4)
Hemoglobin	**7.7**	g/dL	(12.0-17.0)
Mean Cell Volume	78.1	fl	(77.0-91.0)
MCH	25.9	pg	(26.0-32.0)
Mean Platelet Volume	10.5	fl	(6.3-10.2)
Neutrophils	2.8	×10 ^9/L	(2.0-7.0)
Eosinophils	**0.7**	×10 ^9/L	0.02-0.5
Basophils	0.4	×10 ^9/L	(0.02-1.0)
Lymphocytes	1.6	×10 ^9/L	(1.0-3.0)
Monocytes	0.1	×10 ^9/L	(0.2-1.0)
Nucleated Red Blood Cells	0	×10 ^9/L	(0.0-0.0)
**Renal Profile**			
Sodium	**124**	mmol/L	(135-150)
Potassium	4.0	mmol/L	(3.5-5.0)
Urea	**9.9**	mmol/L	(2.5-6.4)
Creatinine	**134**	umol/L	(62-106)
**Liver Function Tests**			
Albumin	**25**	g/L	(35-50)
Total Protein	**56**	g/L	(67-88)
Bilirubin Total	18	umol/L	<23
ALT	**194**	U/L	<44
ALP	**189**	U/L	(32-104)
**Blood Culture Anaerobic**			
Gram negative rods	**3+ Salmonella typhi**		
Sensetivity			
Ceftriaxone	**S**		
Ciprofloxacin	**S**		
Chlorumphenicole	**S**

## Discussion

Lower GI bleeds are rarely as massive and life-threatening as upper GI bleeds, and the need for critical fluid resuscitation is far less common. Resection of terminal ileum carried out for uncontrollable haemorrhage is very rare indeed.

Heavy trichuriasis is more common in children than in adults. This may be due to the unawareness of sanitary procedures and increased incidence of pica which would often lead to an increased worms load. Adult worms do not multiply in the human host, so the number of adult worms per infected person relates to the degree of continued exposure to infective eggs over time [4]. The clinical disease is largely restricted to individuals with a high worm load [5]. Intestinal mucosal ulceration is thought to be due to worm attachment to the intestinal lining since the adult worms got a spear like projection at the anterior end [6]. Hence, the accompanying bacterial infections may have a synergistic effect in the formation of non-specific ulcers.

Trichuriases usually affect the large intestine. In this patient, the terminal ileum was also infected with adult worms. This may be explained by the fact that heavy infection caused spillover of the worms to small intestine.

Intestinal bleeding in typhoid fever usually occurs from the ulcers in the ileum or proximal colon, and the most common colonoscopic manifestations are multiple variable-sized punched-out ulcerations with slightly elevated margin. The combination effect of both *S typhi* and *T. trichiura* led to multiple ulcerations and severe bleeding which caused hypovolemic shock. Ulceration and slough of intestinal mucosa, may suggests concomitant infection with *Entamoeba histolytica*, a well known association [7].

Stool examination was not carried out for this patient throughout the course of investigations. The operation could be avoided if only the surgeon ordered for blood culture and fecal examination in the early stage of management.

## Conclusion

This case illustrates the need for increase awareness among surgeons about infectious causes of lower GI bleeding. Early diagnosis and surgical intervention are essential to minimize high morbidity and mortality.

## Abbreviations

ALP: Alkaline phosphatase
ALT: Alanine aminotransferase
BP: blood pressure
DIVC: disseminated intravascular clotting
GI: gastrointestinal
MCH: mean corpuscular haemoglobin
PR: pulse rate
